# Hemosuccus pancreaticus from a splenic artery pseudoaneurysm as a rare cause of gastrointestinal bleeding: a case report

**DOI:** 10.1097/RC9.0000000000000733

**Published:** 2026-07-22

**Authors:** Siobhan A. Fennell, Salvatore M. Buffa, Ryan Z. Swan, Elizabeth Williams

**Affiliations:** a Department of General Surgery, Zucker School of Medicine at Hofstra/Northwell at Vassar Brothers Medical Center, Poughkeepsie, NY, USA; b Department of Hepatobiliary Surgery, Zucker School of Medicine at Hofstra/Northwell at Vassar Brothers Medical Center, Poughkeepsie, NY, USA; c Department of Gastroenterology, Zucker School of Medicine at Hofstra/Northwell at Vassar Brothers Medical Center, Poughkeepsie, NY, USA

**Keywords:** case report, , hemosuccus pancreaticus, pancreatitis, splenic artery pseudoaneurysm

## Abstract

**Introduction and importance:**

Hemosuccus pancreaticus (HP) is a life-threatening diagnostic challenge due to the relative rarity of the disease. We report a patient with an unknown cause of an upper gastrointestinal bleed (UGI), who was incidentally found to have HP on esophagogastroduodenoscopy (EGD) caused by a large splenic artery pseudoaneurysm. Timely diagnosis and emergent management are required to prevent catastrophic complications.

**Presentation of case:**

An 87-year-old male with a history of alcohol use and recurrent gastrointestinal (GI) bleeds secondary to arteriovenous malformations presented with a GI bleed. Computed tomography angiography (CTA) showed no active hemorrhage but did reveal a 4.0 cm fusiform pseudoaneurysmal dilation of the splenic artery. EGD revealed active bleeding from the ampulla. Due to a high suspicion of a fistulous connection between the pseudoaneurysm and the pancreatic duct, the patient underwent successful embolization.

**Clinical discussion:**

In reported cases of HP secondary to a visceral artery pseudoaneurysm, most patients have a prior diagnosis of acute or chronic pancreatitis. While our patient did have a history of alcohol use, clinically, he exhibited no symptoms or history of pancreatitis, thus contributing to the diagnostic challenge of the case.

**Conclusion:**

A high index of suspicion is required to consider HP as a cause of an acute UGI bleed in a patient with visceral pseudoaneurysms found on imaging, without a prior diagnosis of pancreatitis. These patients have a high risk of mortality and require urgent CTA for timely diagnosis. Management requires multidisciplinary discussion between gastroenterology, hepatobiliary surgery, and interventional radiology, as the two main therapeutic modalities include embolization or surgery.

## Introduction

Gastrointestinal (GI) bleeding is the most common GI disorder requiring hospitalization[1]. Upper GI bleeding (UGIB) describes blood loss from a GI source proximal to the ligament of Treitz, such as the esophagus, stomach, or duodenum, while lower GI bleeding (LGIB) describes a source distal to the ligament of Treitz. The incidence of UGIB is twice as high as that of LGIB in the average population, with an estimated mortality rate of 2–10%[1]. Hemosuccus pancreaticus (HP), described as bleeding from the ampulla of Vater via the pancreatic duct, is a rare and life-threatening cause of UGIB. It has a strong male predilection, with an average age of onset between 50 and 60 years old[2]. It is commonly found in patients with a known history of acute or chronic pancreatitis. Other less common causes include pancreatic tumors, iatrogenic causes from instrumentation manipulation, infectious conditions, and congenital conditions such as pancreatic divisum[2]. There is currently no standard diagnostic protocol or treatment for HP due to the relative rarity of the disease. As it can be a life-threatening condition, once discovered, it is imperative that patients undergo timely resuscitation and management.

HIGHLIGHTS

- Hemosuccus pancreaticus is a life-threatening cause of an upper gastrointestinal bleed.
- Often seen in patients with chronic pancreatitis, but it can also present in those without a previous diagnosis.
- If a visceral artery pseudoaneurysm is present on imaging, one must maintain a high level of suspicion for hemosuccus pancreaticus.
- Multidisciplinary discussion between gastroenterology, hepatobiliary surgery, and interventional radiology is critical for appropriate diagnosis and management.

We present a case of a patient presenting with an acute gastrointestinal hemorrhage who was found to have HP secondary to a large splenic artery pseudoaneurysm. We highlight this patient’s presentation to bring awareness to a relatively rare cause of an UGIB and a diagnostic challenge in a patient without a previous diagnosis of pancreatitis. This case emphasizes the need for timely diagnosis of a rare, life-threatening condition and the necessity for prompt multidisciplinary discussion and treatment. This case report has been reported in line with the SCARE checklist[3].

## Case report

An 87-year-old male with a past medical history of dementia, alcohol use, hypertension, iron deficiency anemia, and a history of recurrent GI bleed in the setting of arteriovenous malformations (AVMs) initially presented as a transfer from an outside community hospital for generalized fatigue and maroon stools. His vitals were BP 147/68, HR 54, RR 16, SpO_2_ 98%, T 96.4°F (oral), and his hemoglobin was 4.8 g/dL (from a baseline of 11 g/dL). He was transfused with 6 units of packed red blood cells and underwent esophagogastroduodenoscopy (EGD), which revealed a small duodenal AVM with oozing into the second portion of the duodenum. This was successfully treated with bipolar electrocoagulation therapy. The remainder of the examination was unremarkable. Simultaneous colonoscopy revealed pancolonic diverticulosis without evidence of bleeding, and an additional non-bleeding AVM in the distal terminal ileum was treated with bipolar electrocoagulation therapy to prevent future bleeding.

He returned to the outside hospital 2 days later after passing a large maroon stool. He underwent a small bowel enteroscopy without evidence of bleeding; however, he was noted to have a hiatal hernia with Cameron ulcer lesions, clotted blood in the gastric fundus, and red blood in the duodenal bulb and proximal jejunum. At the time, as there was no identifiable active source of bleeding, the patient was transferred to a higher level of care for close hemodynamic monitoring.

**Figure 1.**
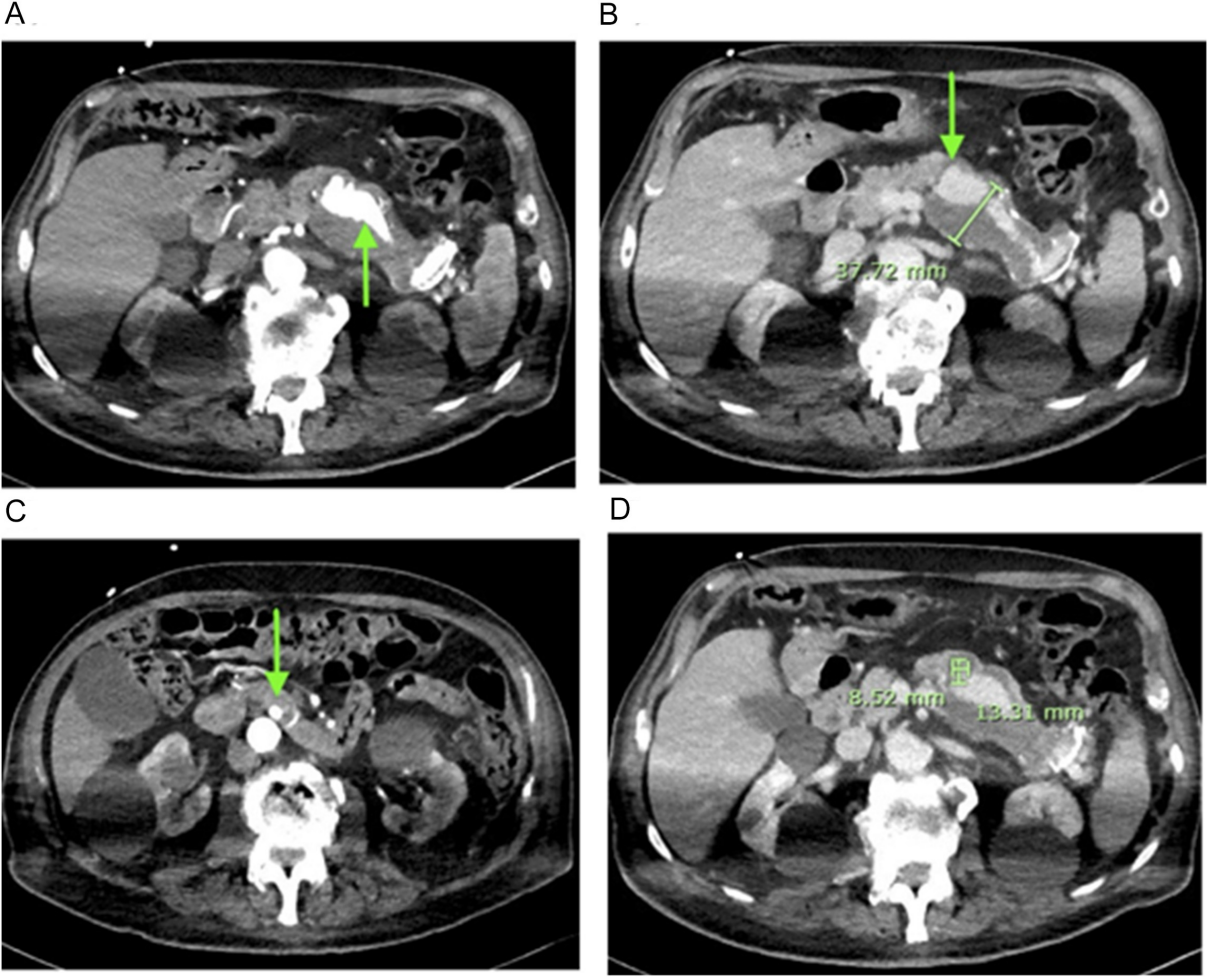
CTA abdomen and pelvis revealing an approximately 4.0 cm fusiform pseudoaneurysmal dilation of the splenic artery (a); an anteriorly projected 13 mm saccular component in the pseudoaneurysm body (b); an 8 mm pseudoaneurysm near the pancreatic uncinate process, likely of the pancreaticoduodenal artery (c); and the 8 mm pseudoaneurysm and 13 mm saccular components with official measurements (d).

Upon arrival, computed tomography angiography (CTA) of the abdomen and pelvis was obtained, which showed no evidence of acute gastrointestinal hemorrhage. Of note, imaging did reveal a 2.5 cm saccular pseudoaneurysm of the common hepatic artery and celiac axis, in addition to a 4.0 cm fusiform pseudoaneurysmal dilation of the splenic artery (Fig. 1a), as well as an anteriorly projected 13 mm saccular component in the body with an 8 mm pseudoaneurysm near the pancreatic uncinate process, likely of the pancreaticoduodenal artery (Fig. 1b).

**Figure 2.**
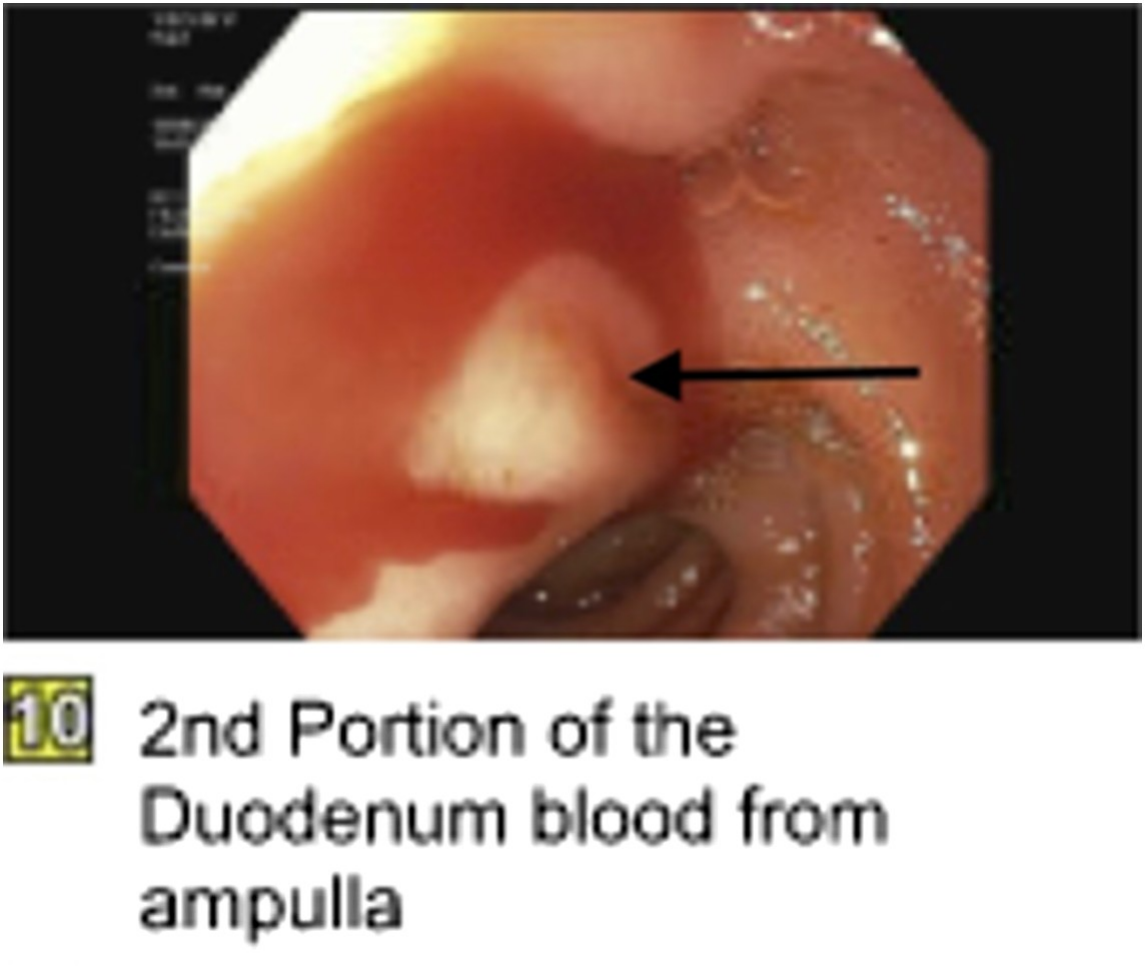
EGD reveals fresh blood in the second portion of the duodenum, with active bleeding extravasating through the ampulla of Vater (arrow indicates the opening of the ampulla).

The patient underwent EGD with findings of a non-bleeding duodenal ulceration in the duodenal bulb, which was injected with 2cc epinephrine submucosally to prevent bleeding. Upon further evaluation, there appeared to be fresh blood in the second portion of the duodenum with active bleeding extravasating through the ampulla of Vater (Fig. 2). A subsequent endoscopic retrograde cholangiopancreatography confirmed filling defects in the pancreatic duct, thought to be secondary to clots. At this time, an urgent consultation with hepatobiliary surgery was placed. On review of the patient’s CTA A/P in conjunction with recent endoscopy findings, there was a high level of suspicion and concern for a fistulous connection to the pancreas with hemorrhage into the pancreatic duct from the large splenic artery pseudoaneurysm abutting much of the pancreatic body and tail.

**Figure 3.**
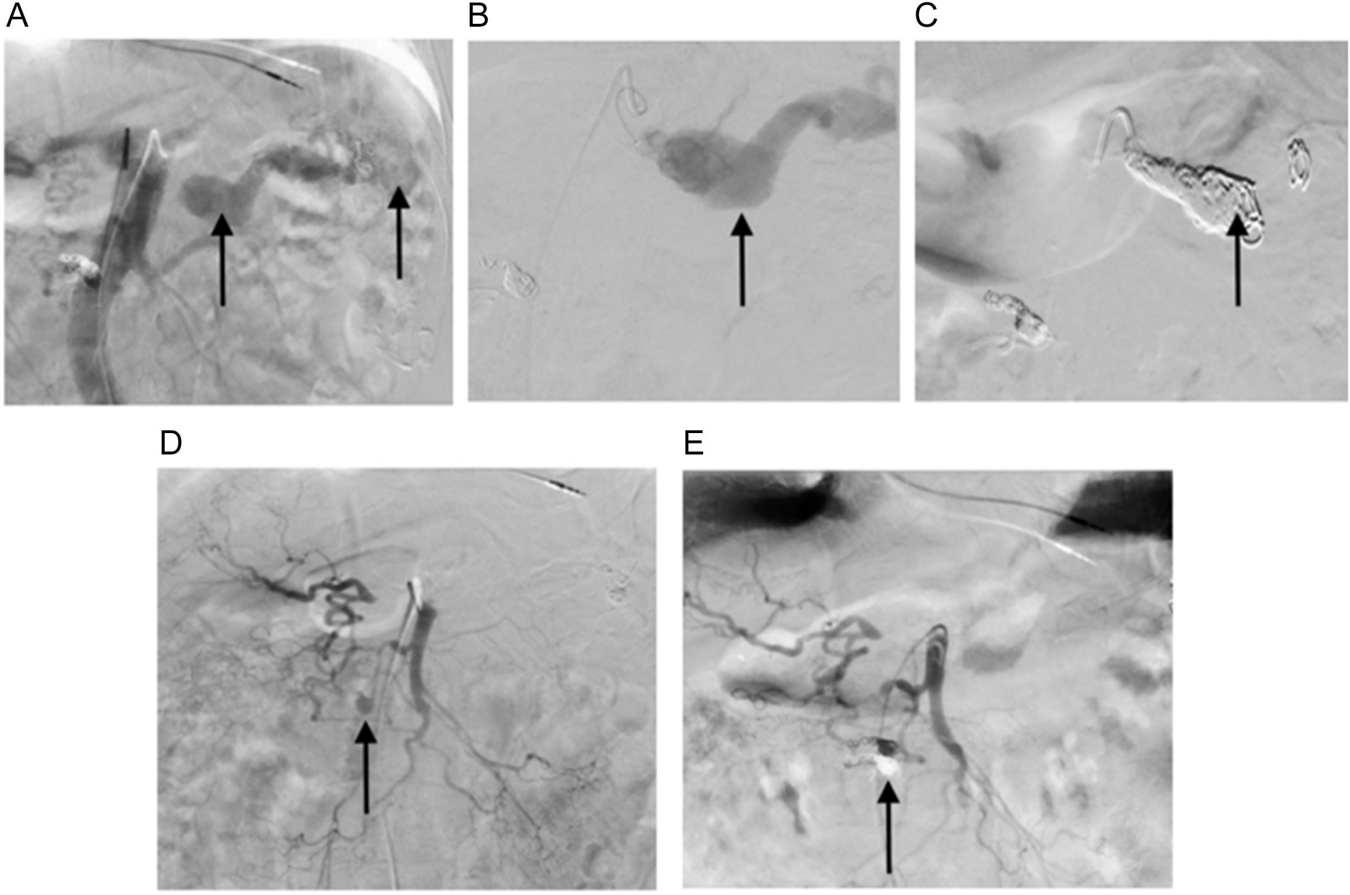
Interventional radiology angiogram identification of the spleen (left arrow) and a 4 cm splenic artery pseudoaneurysm (right arrow) (a, b); successful embolization of the splenic artery pseudoaneurysm (c); identification of an 8 mm posterior inferior pancreaticoduodenal artery pseudoaneurysm on angiogram (d); and successful coil embolization of the posterior inferior pancreaticoduodenal artery (e).

The patient was urgently transferred to interventional radiology, where he underwent fluoroscopically and ultrasound-guided mesenteric arteriography and embolization via right common femoral artery access. The large splenic artery pseudoaneurysm was identified, and pushable and detachable coils were deployed in the proximal splenic artery, followed by cyanoacrylate and Onyx (Fig. 3a–c). The 8 mm pseudoaneurysm of the pancreaticoduodenal artery was also identified, and embolization coils and Onyx were used to embolize the posterior inferior pancreaticoduodenal artery to stasis (Fig. 3d and e).

Following embolization, the patient recovered well in the hospital and was subsequently discharged. He has since followed up in the hepatobiliary surgery clinic and is doing well without any evidence of recurrent bleeding. Given his advanced age and dementia, no further intervention or imaging was recommended unless he begins to develop symptoms.

## Discussion

Visceral artery pseudoaneurysms are relatively rare and often asymptomatic. The splenic artery and hepatic artery are most commonly involved[4]. Pseudoaneurysms, in contrast to true aneurysms, only involve the intima and the media and therefore carry a much higher risk of rupture[4]. Splenic artery pseudoaneurysms are often a complication of recurrent episodes of pancreatitis[4].

First described by Sandbloom in 1970, HP is recognized as a rare cause of upper gastrointestinal bleeding from the ampulla of Vater via the pancreatic duct, with the potential to be aggressive and life-threatening^[^5,6^]^. It is often observed in patients with chronic pancreatitis, with its pathophysiology attributed to the development of a splenic artery pseudoaneurysm secondary to inflammation from acute or chronic pancreatitis, which causes erosion into the pancreatic duct. Pancreatic calcifications, ductal dilation, or irregularity of the pancreatic parenchyma or atrophy are often noted on imaging of individuals with hemosuccus and chronic pancreatitis^[^4,5,7,8^]^. HP secondary to a true splenic artery aneurysm is rare[5].

Predominantly in the literature, patients who present with HP secondary to a splenic artery pseudoaneurysm have a history of acute or chronic pancreatitis^[^4,5,7,8^]^, and cases of patients without a prior diagnosis are infrequently reported in the literature. While our patient did have a history of alcohol use, clinically, he exhibited no symptoms, history, or imaging findings of pancreatitis. *The American Journal of Gastroenterology* published a case similar to ours – HP in a patient with an excessive alcohol use history without a prior diagnosis of acute pancreatitis[9]. However, unlike our patient, imaging findings did show evidence of chronic pancreatitis on both abdominal CT and endoscopic ultrasound[9]. It is likely our patient had undiagnosed chronic pancreatitis secondary to his chronic alcohol use, but the lack of a prior diagnosis contributed to the diagnostic challenges of the case.

There are currently no standardized diagnostic methods for HP due to its complexity and low prevalence. It requires a high index of suspicion based upon the patient’s clinical course and endoscopy/imaging findings^10^. CTA is the recommended standardized diagnostic method when concerned about an UGIB or LGIB, overall demonstrating a 96% sensitivity in patients with a diagnosis of HP[11].

Without prompt recognition, the mortality rate of HP could reach as high as 9.6%, as reported by Yu *et al*^[^12^]^. Presently, current treatment options for HP secondary to splenic artery pseudoaneurysm are not standardized; however, there is a high success rate for control via endovascular intervention[5]. The size of a pseudoaneurysm remains the biggest obstacle. If unsuccessful, urgent surgical intervention with control of the splenic artery pseudoaneurysm and distal pancreatectomy would be curative[5]. The key strength of our case was the prompt transfer from the gastroenterology lab to the interventional radiology suite following discussion between specialties. This led to a very successful outcome and avoidance of major surgery. We place a high emphasis on prompt multidisciplinary discussions to expedite patient care in cases of HP secondary to a splenic pseudoaneurysm.

## Conclusion

In instances of GI bleeding of unknown origin and findings of splenic artery pseudoaneurysm on imaging, a high index of suspicion is required to consider HP as a cause of an acute GI bleed. It is infrequent for a patient to present with HP without a known prior diagnosis of chronic pancreatitis, and acute clinical awareness is imperative. It is often a diagnostic challenge due to the relative rarity of the disease and overall lack of knowledge; therefore, prompt recognition and treatment with interventional radiology or surgical intervention are necessary for successful patient recovery. Multidisciplinary discussion between gastroenterology, hepatobiliary surgery, and interventional radiology is critical for appropriate diagnosis and management.

## Data Availability

All data generated or analyzed during this case report are included in this published article and its accompanying images.

## Meeting presentations

A podium presentation of this case was delivered at the New York Surgical Society’s Interesting Case Night, held at the Penn Club of New York on 2/4/2026.
